# Chlorogenic and phenolic acids are only very weak inhibitors of human salivary α-amylase and rat intestinal maltase activities

**DOI:** 10.1016/j.foodres.2018.07.038

**Published:** 2018-11

**Authors:** Hilda Nyambe-Silavwe, Gary Williamson

**Affiliations:** School of Food Science and Nutrition, University of Leeds, Leeds LS2 9JT, UK

**Keywords:** Amylase, Maltase, Carbohydrates, Type 2 diabetes, Polyphenols, Inhibition, 5-CQA, 5-caffeoylquinic acid, PBS, phosphate-buffered saline, DNS, 3,5-dinitrosalicylic acid

## Abstract

There is increasing evidence that consumption of polyphenol and phenolic-rich foods and beverages have the potential to reduce the risk of developing diabetes type 2, with coffee a dominant example according to epidemiological evidence. One of the proposed mechanisms of action is the inhibition of carbohydrate-digesting enzymes leading to attenuated post-prandial blood glucose concentrations, as exemplified by the anti-diabetic drug, acarbose. We determined if the phenolic, 5-caffeoylquinic acid, present in coffee, apples, potatoes, artichokes and prunes, for example, and also selected free phenolic acids (ferulic acid, caffeic acid and 3,4-dimethoxycinnamic acid), could inhibit human salivary α-amylase and rat intestinal maltase activities, digestive enzymes involved in the degradation of starch and malto-oligosaccharides. Using validated assays, we show that phenolic acids, both free and linked to quinic acid, are poor inhibitors of these enzymes, despite several publications that claim otherwise. 5-CQA inhibited human α-amylase only by <20% at 5 mM, with even less inhibition of rat intestinal maltase. The most effective inhibition was with 3,4-dimethoxycinnamic acid (plateau at maximum 32% inhibition of human α-amylase at 0.6 mM), but this compound is found in coffee in the free form only at very low concentrations. Espresso coffee contains the highest levels of 5-CQA among all commonly consumed foods and beverages with a typical concentration of ~5 mM, and much lower levels of free phenolic acids. We therefore conclude that inhibition of carbohydrate-digesting enzymes by chlorogenic or phenolic acids from any food or beverage is unlikely to be sufficient to modify post-prandial glycaemia, and so is unlikely to be the mechanism by which chlorogenic acid-rich foods and beverages such as coffee can reduce the risk of developing type 2 diabetes.

## Highlights

• Chlorogenic and phenolic acids weakly inhibit human α-amylase activity.
• Chlorogenic acids weakly inhibit rat maltase activity.
• Free phenolic acids exhibit no inhibition of rat maltase activity.

## Graphical abstract

**Figure:**
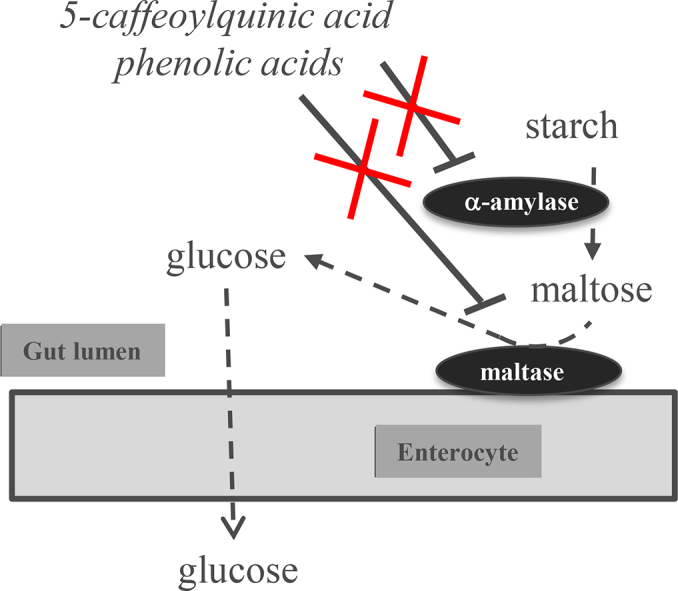

## 1 Introduction

**Fig. 1:**
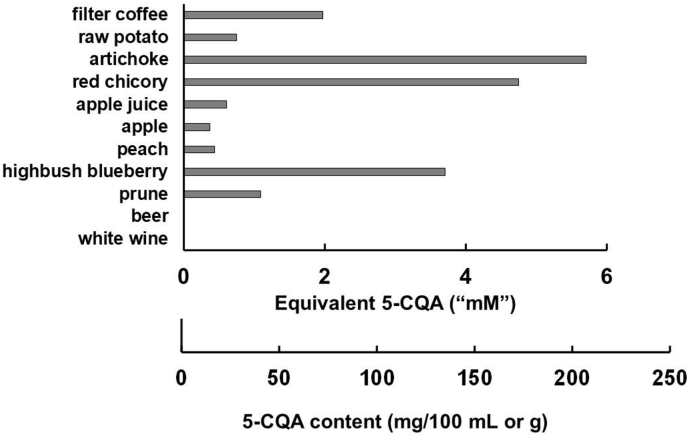
Content of 5-caffeoylquinic acid in various foods and beverages. Data is obtained from phenol-explorer (Neveu et al., 2010), as mg per 100 mL or 100 g. For ease of assessment, the value has been converted into apparent "mM" based on the content. This is close to the correct value for beverages, but for foods would depend on chewing, extraction and other parameters, and is given as a guide only.

Phenolic acids occur at high levels in many foods, including coffee, apples, potatoes, artichokes and prunes, and are predominantly found in the form of chlorogenic acids, where the phenolic acid moiety is attached to a quinic acid to form various isomers (Clifford, 1999). In foods, the most abundant is 5-caffeoyl-quinic acid (IUPAC numbering; 5-CQA, Fig. 1) and this isoform has also been the most studied. Numerous papers and reviews have been published on the potential health effects of phenolic acids (Van Dam & Hu, 2005; Higdon & Frei, 2006). In coffee drinkers, by far the most common source of chlorogenic acids in the diet is coffee (Clifford, 2000), since it is both one of the richest foods and beverages, and is also consumed widely and abundantly in many countries worldwide (Higdon & Frei, 2006).

Coffee could be a good dietary intervention for risk reduction for developing type 2 diabetes, as consumption has been linked to a reduced risk of developing the disease in a dose-dependent manner (Ding, Bhupathiraju, Chen, van Dam, & Hu, 2014). A recent review (Santos & Lima, 2016) and a systematic review (Van Dam & Hu, 2005) strongly support this hypothesis, in addition to studies in The Netherlands (Van Dam & Feskens, 2002) and Finland (Tuomilehto, Hu, Bidel, Lindström, & Jousilahti, 2004). However, the mechanism of action by which coffee confers this antidiabetic effect is not very clear (Van Dam & Hu, 2005), but among the mechanisms proposed is attenuation of carbohydrate digestion, as suggested for other polyphenols (Hanhineva et al., 2010; Williamson, 2013). In this respect, several *in vitro* studies (Karim, Holmes, & Orfila, 2017; Narita & Inouye, 2011; Oboh, Agunloye, Adefegha, Akinyemi, & Ademiluyi, 2015) have reported that coffee phenolics may have comparable effects to the drug acarbose, *i.e.* inhibition of α-amylase and α-glucosidase digestive activities. Chlorogenic acid (specifically 5-CQA) and caffeic acid were alleged to be inhibitors of both α-amylase (porcine) and α-glucosidase enzymes with IC_50_ values <100 μM (Oboh et al., 2015). Two other studies showed that porcine α-amylase was inhibited by 5-CQA with IC_50_ values also <100 μM (Karim et al., 2017; Narita & Inouye, 2009) with caffeic and quinic acid being weaker inhibitors with IC_50_ values of >0.3 and > 25 mM respectively. However most of the studies used porcine, not human, α-amylase. Inhibition of different sources of enzyme varies widely (Nyambe-Silavwe et al., 2015; Pyner, Nyambe-Silavwe, & Williamson, 2017) and hence the current study aimed at using α-amylase from humans (salivary α-amylase) to reassess this activity of phenolic acids. We also determined the effects on α-glucosidase using a rat intestinal extract as the enzyme source, which has comparable inhibition properties to the human intestinal enzyme (Pyner et al., 2017).

## 2 Materials and methods

### 2.1 Reagents and standards

Caffeic acid, ferulic acid, 3,4-dimethoxycinnamic acid, 5-caffeoyquinic acid, 3,5-dinitrosalicylic acid, potassium sodium tartrate, amylose and human salivary α-amylase type IX-A were all purchased from Sigma-Aldrich. Co., Ltd., Dorset, UK. Oasis MAX cartridge 1 mL (30 mg) and 3 mL (60 mg) were purchased from Waters Ltd., Milford, MA, U.S.A. All the reagents were of the highest purity and standards were ≥98 %. The colour reagent was prepared by mixing 20 mL of 96 mM of 3,5-dinitrosalicylic acid with 8 mL of 5.3 M (12 g in 8 mL of 2 M sodium hydroxide) and 12 mL Millipore water. Human salivary amylase type IX-A stock concentration of 1.25 U/mL was prepared in PBS (0.01 M, pH 6.9) to give 0.5 U/mL in the assay according to the optimized assay (Nyambe-Silavwe et al., 2015).

### 2.2 α-Amylase inhibition assay

Amylose (1 mg/mL) was used as the substrate and the assay was conducted according to the optimized assay (Nyambe-Silavwe et al., 2015). A total assay volume of 500 μL was used consisting of 200 μL each of amylose and enzyme, 50 μL PBS and 50 μL of potential inhibitor at different concentrations. The potential inhibitor was replaced by an equal volume of PBS for the control. The reaction was carried out at 37 °C for 10 min upon addition of 200 μL of pre-incubated enzyme at 37 °C to a mixture of substrate, PBS and varying concentrations of inhibitor, also pre-incubated at 37 °C. To end the reaction, the samples were placed in the water bath at 100 °C for 10 min, cooled on ice and centrifuged for 5 min. Solid phase extraction (SPE) was carried out on the sample for removal of polyphenols that have been shown to interfere with the colour reagent solution containing 3,5-dinitrosalicylic acid (DNS). DNS reagent was added to the sample in a ratio of 2:1 and heated at 100 °C for 10 min. From each sample, 250 μL was placed in a 96 well plate and the absorbance was recorded at 540 nm. The rate of enzyme inhibition was calculated as a percentage of the control (without inhibitor) using the formula:

% Inhibition = ((Abs Control − Abs sample)/Abs control) x 100.

Where inhibition was obtained above 50%, IC_50_ was calculated graphically by dose-dependent inhibition.

### 2.3 α-Glucosidase inhibition assay

The method used to assess rat α-glucosidase inhibition was adapted from Adisakwattana, Charoenlertkul, & Yibchok-anun, 2009 as modified by Nyambe-Silavwe & Williamson, 2016. An assay volume of 500 μL was used and consisted of 50 μL of sodium phosphate buffer (10 mM, pH 7), 50 μL of potential inhibitor, 200 μL of acetone-derived protein intestinal extract from rat intestine (4 mg solid/mL for maltose) and 200 μL of substrate (3 mM maltose) (Nyambe-Silavwe & Williamson, 2016). Sodium phosphate buffer (50 μL) was put in place of the potential inhibitor for the control sample. The reaction was carried out at 37 °C for 20 min by adding the enzyme source to a mixture of sodium phosphate buffer, potential inhibitor and substrate. The reaction was stopped by heating in a water bath at 100 °C for 10 min, cooled to room temperature, polyphenols removed by solid phase extraction, hexokinase reagent added and absorbance read at 340 nm in a plate reader. Inhibition in the samples was calculated as a percentage of the control.

### 2.4 Statistical analysis

Statistical analysis was performed by one-way analysis of variance using the Number Cruncher Statistical System version 6.0 software (NCSS, LLC). Significant differences were assessed with Tukey-Kramer multiple comparison test (*p* ≤ .05). The data are expressed as the mean ± standard deviation (*n* = 3).

## 3 Results

### 3.1 Inhibitory effect on human salivary α-amylase and rat maltase activity

**Fig. 2:**
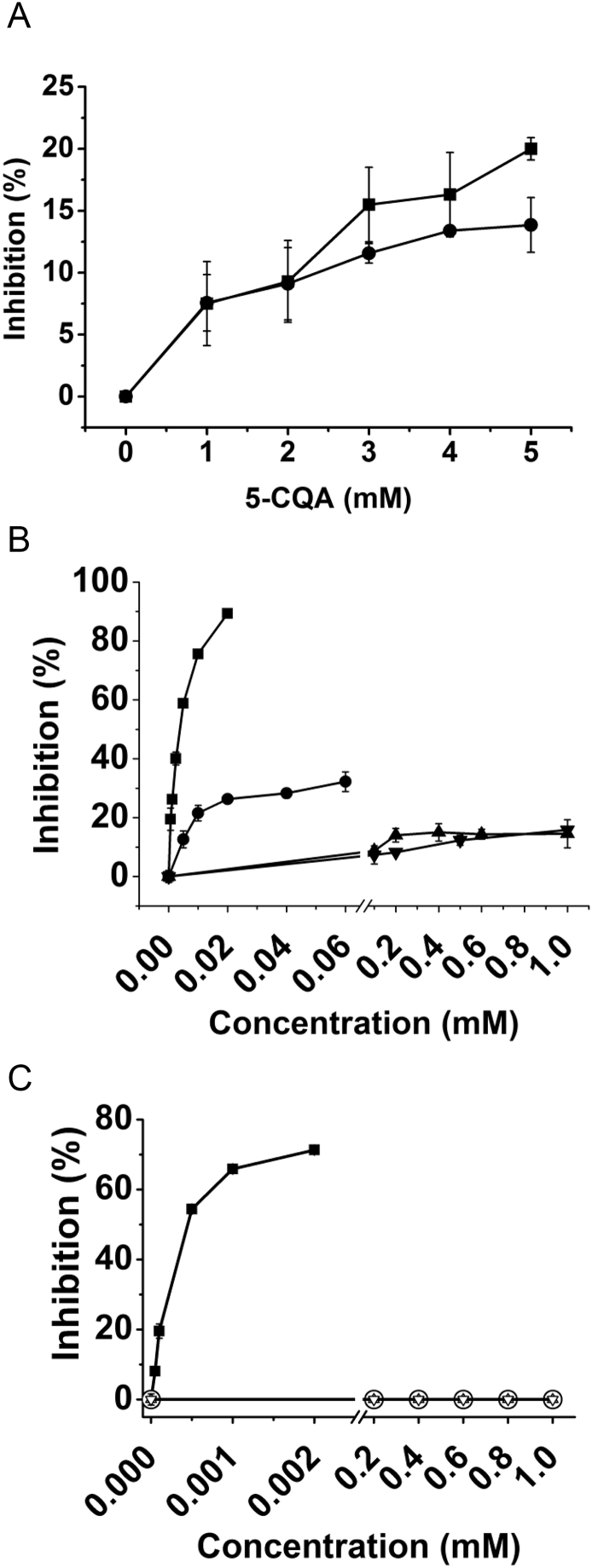
Inhibition of enzyme activities by phenolics. A: Inhibition of α-amylase (■) and of rat intestinal maltase (●) activities by 5-caffeoylquinic acid (5-CQA). B: Inhibition of α-amylase activity using amylose as substrate by acarbose (■), 3,4-dimethoxycinnamic acid (●), caffeic acid (▲) and ferulic acid (▼). C: Inhibition of rat intestinal maltase activity using maltose as substrate by acarbose (■),3,4-dimethoxycinnamic acid (○), caffeic acid (∆), and ferulic acid (◊).

5-CQA only weakly inhibited human salivary α-amylase and rat intestinal α-glucosidase activities with maximum of 20.5 and 13.9% respectively at the highest (5 mM) tested concentration (Fig. 2A). As a positive control, acarbose (a well-known carbohydrase inhibitor) exhibited an IC_50_ value of 3.5 ± 0.3 μM for human salivary α-amylase and 0.40 ± 0.01 μM for rat intestinal maltase activities respectively (Fig. 2B and C), as expected (Nyambe-Silavwe et al., 2015).

Against human salivary α-amylase, ferulic acid, caffeic acid and 3,4-dimethoxycinnamic acid showed some dose-dependent inhibition, but the extent of inhibition was very low compared to acarbose (Fig. 2B). The most effective was 3,4-dimethoxycinnamic acid which gave maximum inhibition of 32% at 0.6 mM, but with no further change at increasing concentrations (*p* ≥ .05). Caffeic acid and ferulic acid both showed inhibition of <20% at the highest concentration tested of 1 mM. None of the free phenolic acids inhibited rat intestinal maltase activity (Fig. 2C) even at the highest concentration tested.

## 4 Discussion

The aim of the present study was to determine whether the antidiabetic properties attributed to drinking coffee (and possibly to other foods or beverages containing chlorogenic acids) are due to an acarbose-like action, a drug used to attenuate hyperglycaemia through inhibition of carbohydrate-digesting enzymes. In a detailed survey of espresso coffees, the strongest form of coffee normally consumed and commercially available in a major city of the UK, a typical serving of espresso was in the range of 24–422 mg per serving in an average serving size of 43 mL (Crozier, Stalmach, Lean, & Crozier, 2012). Since 5-CQA was on average 51% of the total content, then this corresponds to a 5-CQA concentration of 4.8 mM. This is diluted in the mouth with saliva and in the intestine with various digestive juices (Williamson, 2013). At this concentration, we would predict <20% inhibition of human salivary α-amylase, which is not sufficient to exert an effect *in vivo* on carbohydrate digestion and post-prandial glucose concentrations, since we previously found that oleuropein, with IC_50_ values superior to 5-CQA (0.56 mM for rat intestinal α-glucosidase and 1.4 mM for human salivary α-amylase), did not attenuate post-pranidal blood glucose after consumption of bread as a carbohydrate-rich food (Kerimi et al., 2018). According to phenol-explorer (Neveu et al., 2010), filter coffee contains considerably less 5-CQA (Fig. 1), at ~2 mM. In addition, for most other commonly consumed foods, the concentration of chlorogenic acid is lower or much lower than in coffee (Fig. 1). Raw potato, for example, does not contain enough chlorogenic acid to exert any significant inhibition of α-amylase, and after cooking, the amount decreases; for oven baking or French fries, all chlorogenic acid is lost, for boiled potatoes, only 35% is left, or 55% after microwaving (Dao & Friedman, 1992). Free phenolic acids are present at very low levels in coffee (Encarnação, Farrell, Ryder, Kraut, & Williamson, 2015) and in most other foods (Neveu et al., 2010).

We found here that 5-CQA and free phenolic acids are very weak inhibitors of human salivary α-amylase, and even at high concentrations, 25% inhibition was generally not reached. We have specifically used a naturally-occurring substrate (amylose from starch) rather than a synthesized dye-linked substrate, which would have markedly different affinity for the enzyme, have also used a human source of α-amylase, and have ensured that the phenolic acids do not interfere in the DNS product determination. Several studies using the porcine pancreatic enzyme (Funke & Melzig, 2005; Karim et al., 2017; Narita & Inouye, 2009; Narita & Inouye, 2011; Oboh et al., 2015) have reported that chlorogenic acids inhibited α-amylase, and obtained an IC_50_ value of 0.08 mM for 5-CQA (Narita & Inouye, 2011) and of 0.026 mM for caffeic acid (Oboh et al., 2015). It is now well established (Nyambe-Silavwe et al., 2015; Pyner et al., 2017) that the use of different enzyme sources for the inhibition assays as well as different substrates (Nyambe-Silavwe et al., 2015) can yield very different results.

For inhibition of rat intestinal maltase, 5-CQA was a weak inhibitor, but none of the free phenolics showed inhibition. Other research ([Bibr bb0060][Bibr bb0075]) also reported inhibition of α-glucosidase which is in contrast to our results demonstrating minimal inhibition. Human maltase is less susceptible to inhibition than rat maltase (Pyner et al., 2017), and hence we would have expected even lower inhibition in volunteers *in vivo*. We therefore conclude that the anti-diabetic effects of coffee consumption are not due to inhibition of carbohydrate-hydrolysing enzymes. However, several studies have shown that consumption of coffee reduces postprandial blood glucose levels. There was a significant reduction in total area under the glucose curve in a rat model after consumption of a standardised meal containing carbohydrate with chlorogenic acid (Tunnicliffe, Eller, Reimer, Hittel, & Shearer, 2011). In healthy males, it was shown that consumption of coffee polyphenol extract significantly reduced peak postprandial blood glucose as well as improving postprandial blood GLP-1 response which is associated with anti-diabetic effects (Jokura, Watanabe, Umeda, Hase, & Shimotoyodome, 2015). It was also shown, both in humans (Sarriá, Martínez-López, Mateos, & Bravo-Clemente, 2016) and in a rat model (Budryn et al., 2017), that consumption of green/roasted coffee blend led to lowering of blood glucose. Hence due to overwhelming evidence that coffee and its phenolic acids are associated with antidiabetic properties *via* modulation of glucose metabolism, other mechanisms may be involved which include inhibition of intestinal glucose transport. In this respect, when consumed with a glucose bolus, coffee exhibited some effect on post-prandial glycemia, although not directly on plasma glucose concentration (Johnston, Clifford, & Morgan, 2003), and this could be at least partly due to effects on glucose transporters or on hormonal response to food. These aspects should be addressed in future studies.
